# Management of Ectropion Associated With a Malunited Zygomaticomaxillary Complex Fracture: A Case Report

**DOI:** 10.7759/cureus.52909

**Published:** 2024-01-25

**Authors:** Deepankar Shukla, Nitin Bhola

**Affiliations:** 1 Oral and Maxillofacial Surgery, Sharad Pawar Dental College and Hospital, Datta Meghe Institute of Higher Education & Research, Wardha, IND

**Keywords:** malunion, z-plasty, temporalis muscle graft, ankylotic mass, ectropion

## Abstract

We report the case of a 35-year-old male patient who presented with a right zygomaticomaxillary complex fracture, which was five months old. It was associated with ectropion over the right eye. Diagnosis was made by clinical examination and confirmed by computed tomography, which included a three-dimensional reconstruction view. The patient was concerned about a projecting deformity over the right side of his face and blurring of vision. Surgical rationale of treatment was to easily access the surgical site for the correction of deformity and to achieve the desired facial contour and ectropion correction with uneventful postoperative healing. Deformity at the right zygomatic arch was exposed by a hemicoronal incision. Ectropion over the lower eyelid was addressed by performing Z-plasty. Outcomes were esthetically pleasing with no loss of motor and sensory functions loss. The patient was followed up for six months.

## Introduction

The zygomaticomaxillary complex determines the breadth of the midface and accentuates the prominence of the cheek, serving as a buttress for the face and functioning as the basis of one's aesthetic look [1]. The inferior and lateral orbital rims, along with the anterior and posterior maxillary sinus walls and the zygomatic arch, make up the zygomaticomaxillary complex. Establishing an optimal projection can be difficult because of the complexity of the structural design [2]. Ectropion is a condition characterized by the outward turning or eversion of the eyelid, occurring more commonly in the lower eyelid. It could be either bilateral or unilateral and can be acquired or congenital. The four forms of acquired ectropion include involutional, paralytic, cicatricial, and mechanical [3]. The anterior lamella of the eyelid is vertically shortened and/or scarred in cicatricial lower lid ectropion and can be caused by chemical burns, surgical or mechanical trauma, pharmaceuticals, chronic inflammation, sun damage, or involutional changes [4].

## Case presentation

**Figure 1:**
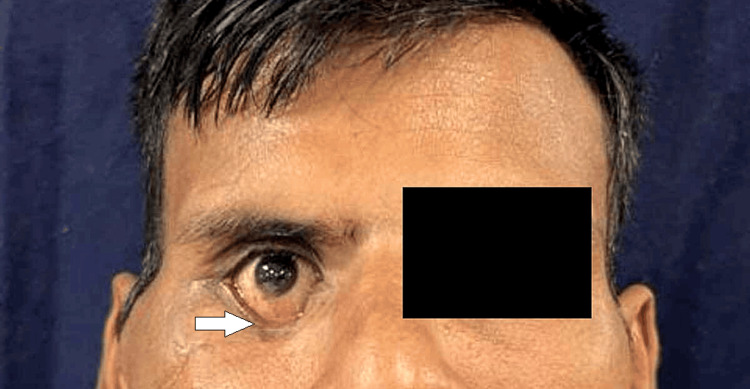
Preoperative photograph (frontal view).

A 35-year-old male reported to our facility with a chief complaint of poor aesthetics over the right side of the face and reduced mouth opening for the last four months. The patient revealed a history of motor vehicle accidents due to a bike five months prior to presentation. The patient was under the influence of alcohol at the time of accident. There was no history of loss of consciousness, seizure or convulsion episodes, or vomiting. The patient was admitted to the neurosurgery ward at our facility to manage fractures of the C1 and C2 vertebrae. On clinical examination, the face was asymmetrical due to a malunited zygomatic arch seen over the right side. A scar was seen over the right side of the lower eyelid (Figure 1). Blurred vision in the downward and medial gaze was seen in the right eye. Rest assured, all gazes were intact. Watering from the right eye was present. Grade IV facial nerve weakness, according to the House-Brackmann classification (post-injury), was present due to the typical characteristic features of obvious asymmetry and incomplete eye closure. On palpation, all findings of previous examinations were confirmed. On temporomandibular joint examination, there was restricted jaw movements with no deviation and no clicking, as well as reduced mouth opening of approximately 10 mm.

**Figure 2:**
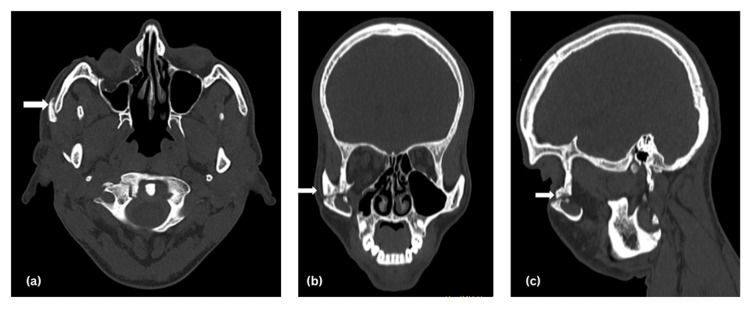
(a) Axial, (b) coronal, and (c) sagittal views of head CT showing the malunited zygomaticomaxillary complex fracture.

**Figure 3:**
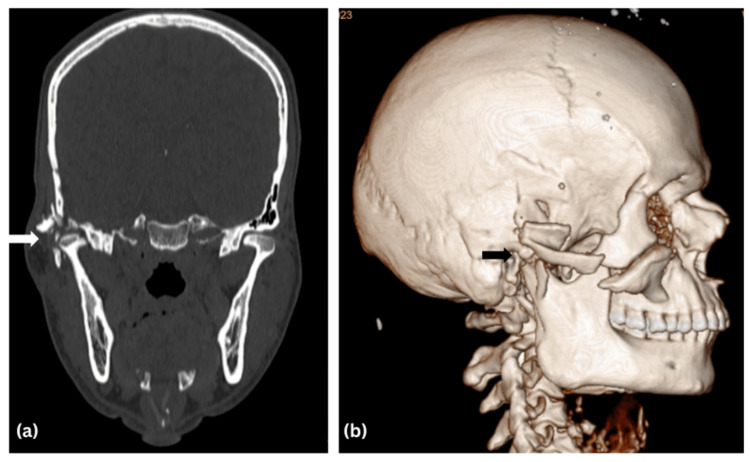
(a) Coronal view and (b) three-dimensional reconstruction of head CT showing the ankylotic mass over the right temporomandibular joint.

Step deformity was present over the right zygomatic arch and infraorbital rim. On intraoral examination, all sets of permanent teeth were present with bilaterally stable occlusion. Provisional diagnosis of malunited right zygomaticomaxillary complex fracture with ectropion with House-Brackmann grade IV facial nerve weakness was made. Radiological investigation included a CT scan of the head and face with three-dimensional reconstruction (Figures 2, 3). The CT revealed a malunited right zygomatic arch fracture along with a right zygomaticomaxillary buttress fracture, and an ankylotic mass over the right temporomandibular joint.

**Figure 4:**
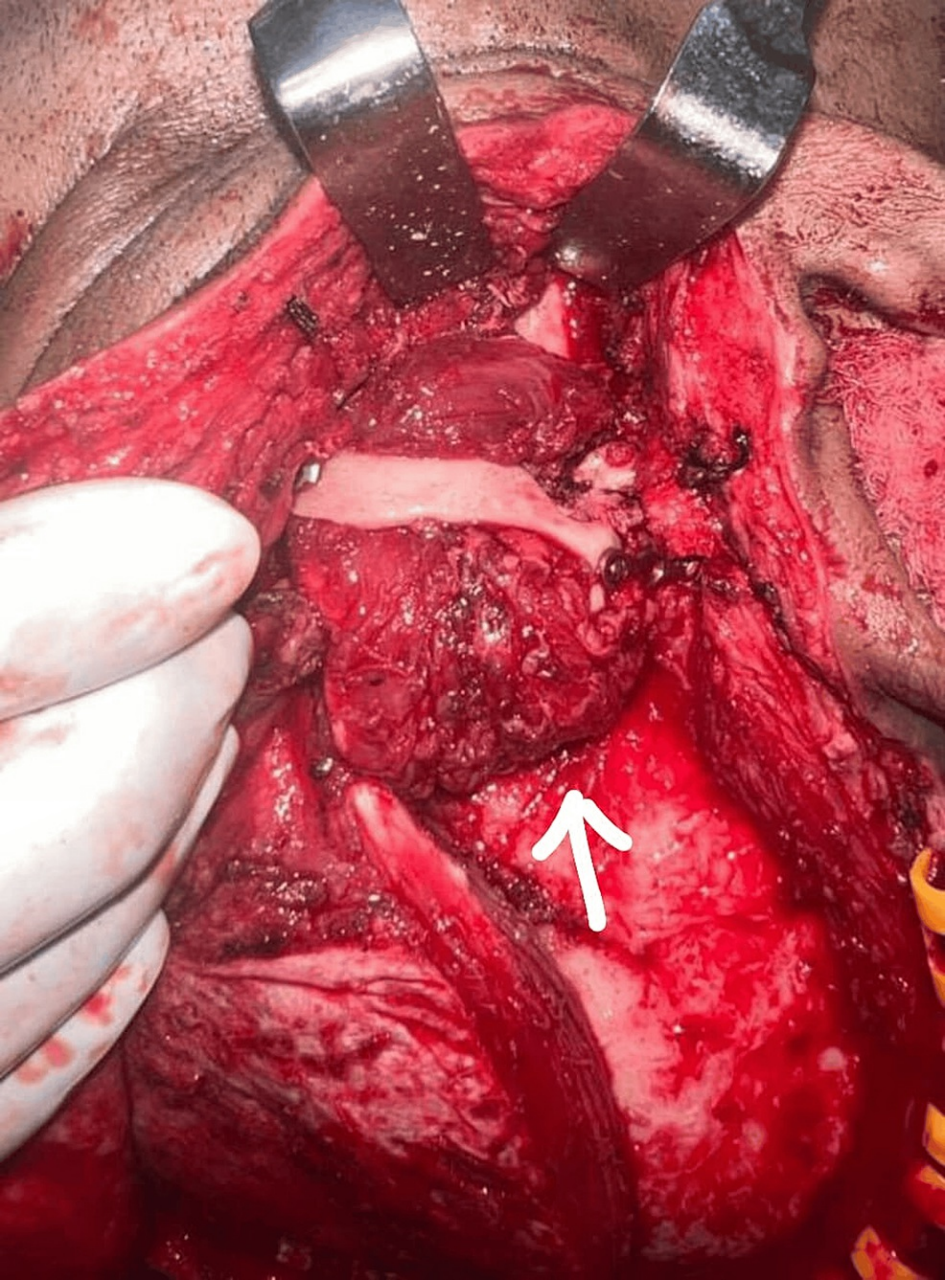
Interpositional gap arthroplasty performed using the temporalis muscle.

**Figure 5:**
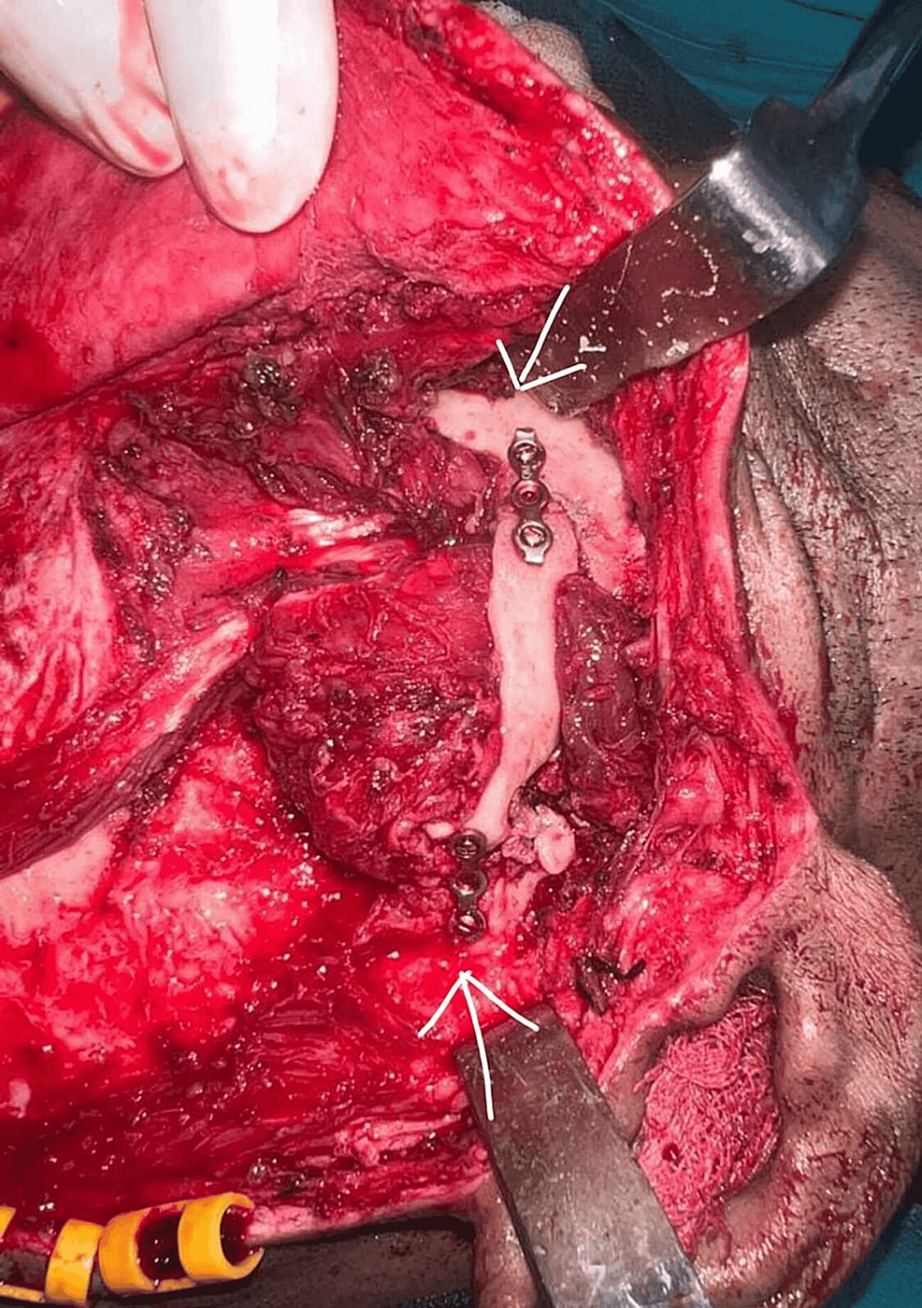
Plating of the right zygomatic arch.

The final diagnosis made from the examination and palpatory findings was that of a malunited right zygomaticomaxillary complex fracture with trismus and ectropion of the right eye. Surgical intervention was planned for this case under general anesthesia. The approach used for surgical intervention was hemicoronal incision over the right side. A coronoidotomy was performed on the right side, following which the mouth opening achieved was 30 mm. Also, a bony ankylotic mass was seen over the right side of the TMJ following trauma; its exposure was followed by the release of the ankylotic mass over the right side following interpositional gap arthroplasty with temporalis muscle graft of the same side (Figure 4). The arch was plated using a 1 x 1.5 mm three-hole continuous titanium plate with two screws of 1.5 x 6 mm each (Figure 5).

Standard marking was done for maxillary vestibular incision from region 13 to region 17, and the incision was given and deepened through mucosa and muscle. A subperiosteal dissection was performed to expose the bone over the right zygomatic buttress region. A surgical osteotomy was performed over the right zygomatic arch region, the right frontozygomatic region, and the right zygomatic buttress region by a high-speed bur while under copious irrigation to avoid necrosis of the bone. Bone fragment was harvested from the right zygomatic buttress region.

**Figure 6:**
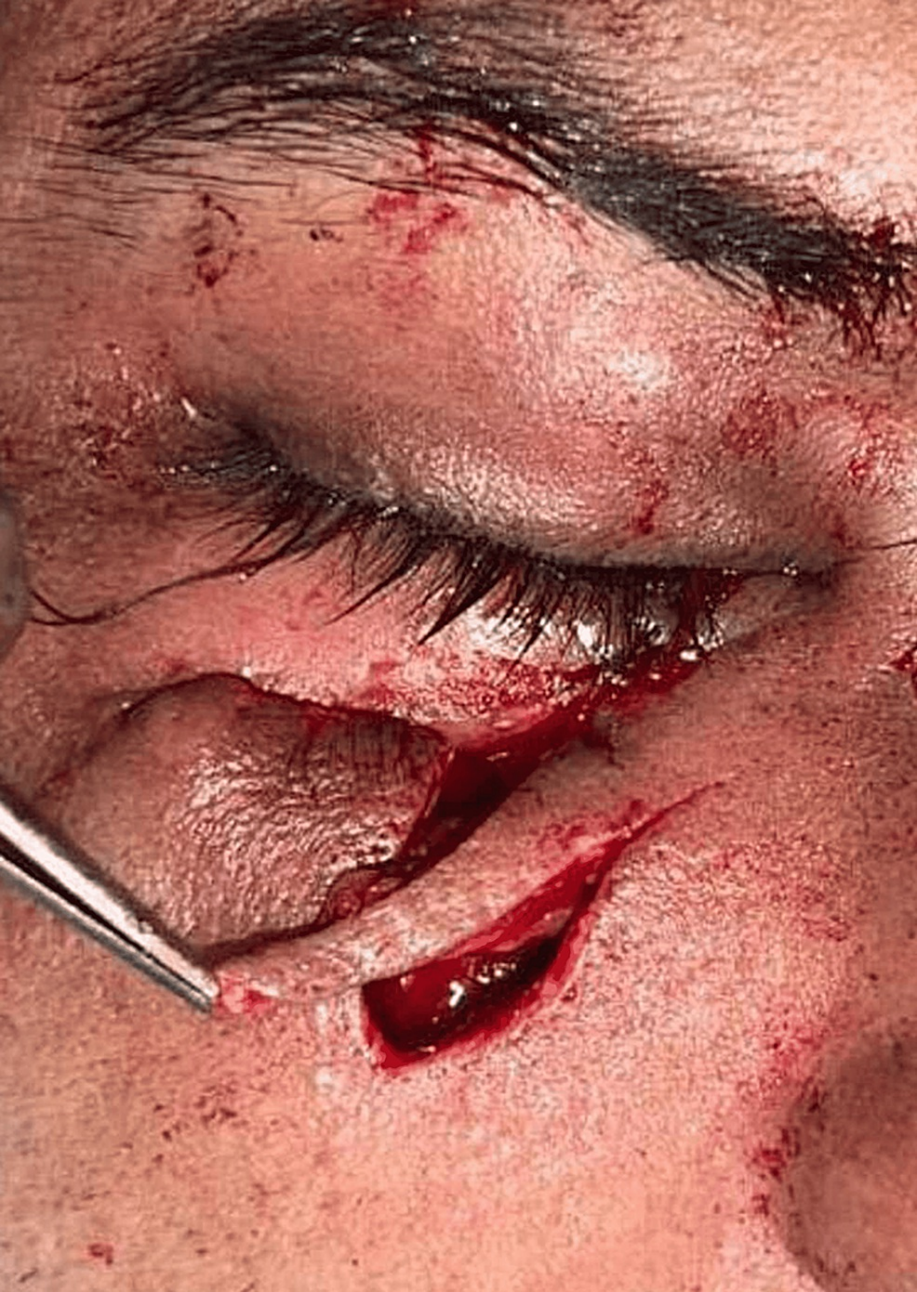
Z-plasty of ectropion of the right eye.

**Figure 7:**
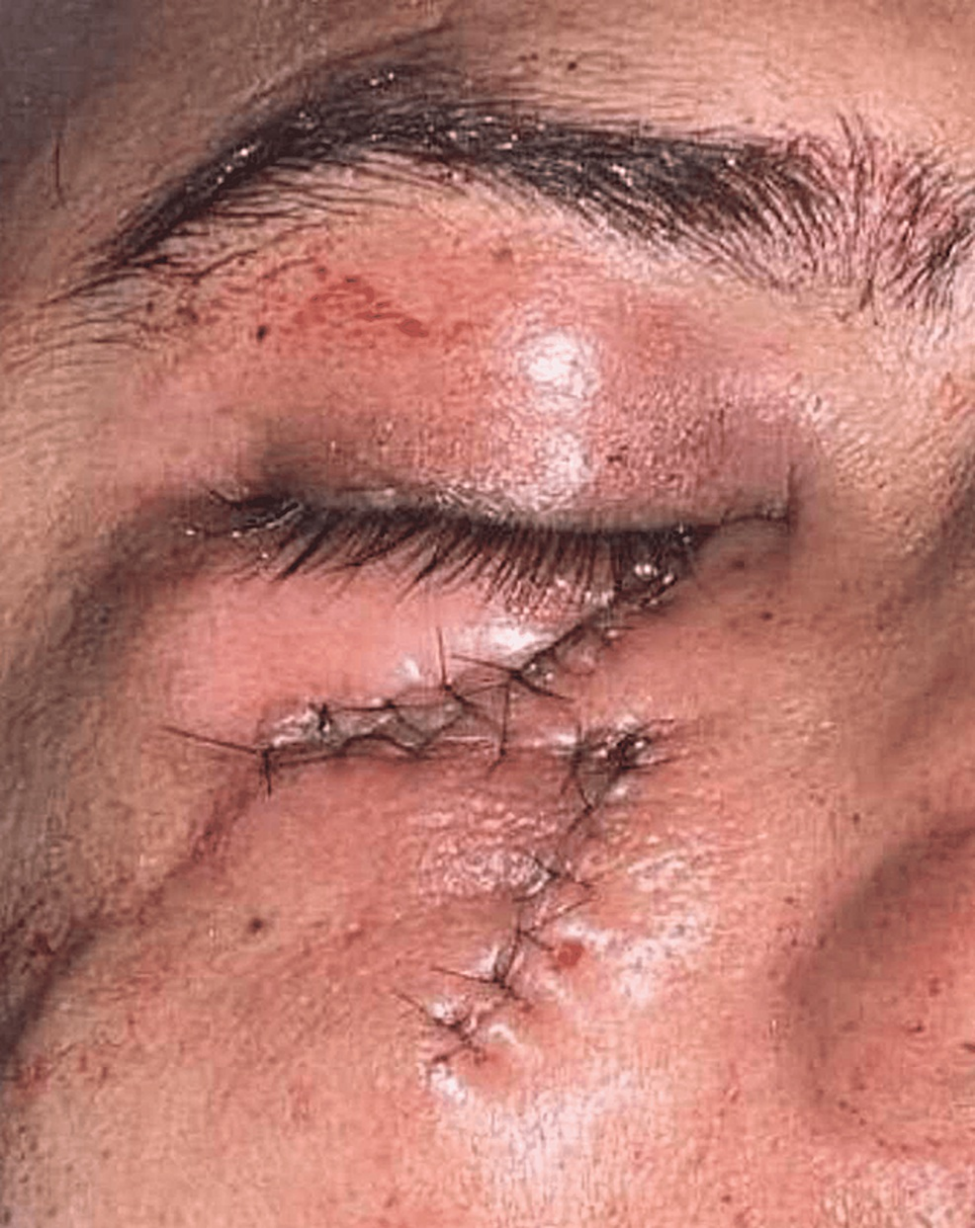
Closure of Z-plasty of ectropion of the right eye.

The infraorbital rim was reconstructed with the free bone graft taken from the right zygomatic buttress region. Bone fragments were reapproximated, and reduction was performed. Fixation was done using a 1 x 1.5 mm, five-hole continuous titanium plate with three screws of 1.5 x 6 mm each. Ectropion over the lower eyelid of the right eye was addressed, for which Z-plasty was performed (Figures 6, 7).

 

**Figure 8:**
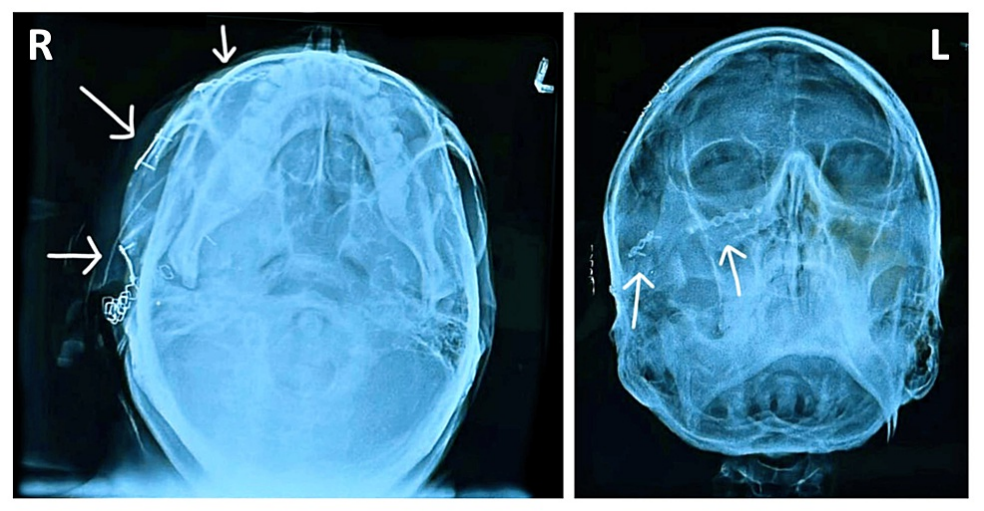
Postoperative radiographs showing fixation (SMV and PNS views). SMV, submentovertex; PNS, paranasal sinus

**Figure 9:**
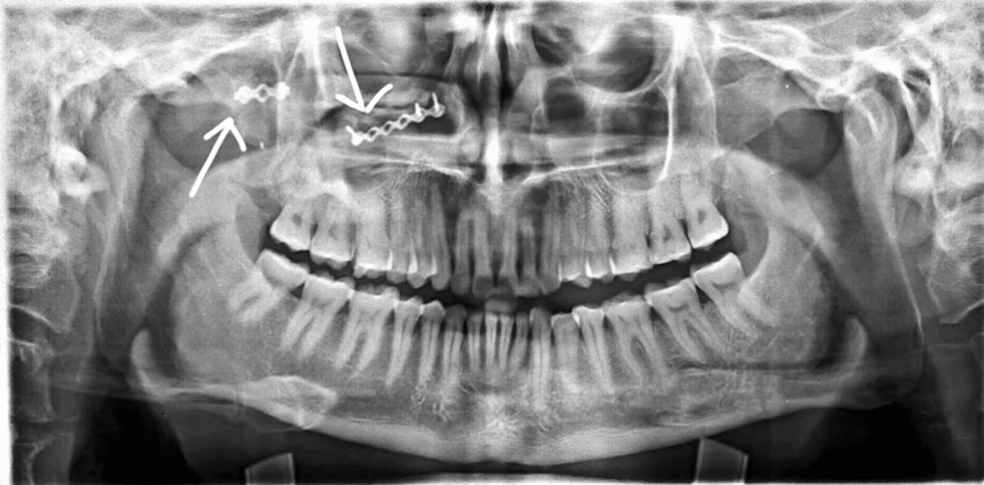
Postoperative radiograph showing fixation.

A Mini Vac drain was placed over the right preauricular region, after which closure was performed in layers: intraorally with 3-0 Vicryl, extraorally for muscle and periosteum with 3-0 Vicryl, and skin with 4-0 Prolene. Skin stains over the right temporal region were given. E/O pressure dressing was given over the right preauricular region. Postoperatively, the patient was advised to apply pressure dressings over the temporal and preauricular regions. Also, the application of ciprofloxacin 0.3% ointment over the sutured site was advised. Postoperatively, the patient was advised to take steroids in tapering dosages, and tablet Neurobion Forte, twice daily, was advised for facial nerve weakness. Figure 8 shows the postoperative submentovertex view and paranasal sinus view, and Figure 9 shows the postoperative orthopantomogram.

**Figure 10:**
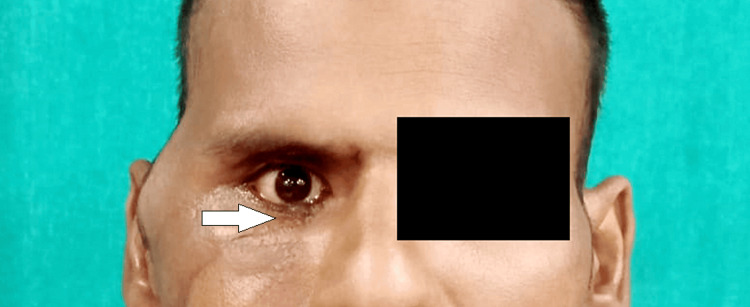
Follow-up photograph (frontal view).

The patient was called after five days for follow-up, and further evaluation was conducted. Surgical site was healthy and healed satisfactorily. There were no signs of gapping or dehiscence, bleeding, or pus discharge. Figure 10 shows the image after suture removal.

The patient had persistent facial nerve weakness for which the patient was advised to continue tablet Neurobion twice daily. Also, the patient had persistently reduced mouth opening, for which active mouth opening was advised.

## Discussion

Zygomatic malunion can result from incorrect fixation, reduction, or nonintervention when surgery is recommended. In some cases, the last scenario may occur when the patient's overall health restricts the possibility of immediate surgical intervention, when the patient chooses to delay seeking treatment, or when the patient refuses surgery altogether. The indicators and symptoms, including enophthalmos, depression of the malar prominence, restricted mandibular motion, and altered pupillary level, mirror those observed in a person recently afflicted by a fracture of the zygoma [5]. Secondary correction of the zygomaticomaxillary complex comes with its own inherent challenges [6]. It is a difficult and challenging task to reposition a significantly malpositioned zygoma back to its correct place while simultaneously repairing preexisting orbital abnormalities.

Fixation is always required. A number of techniques have been used in the past for zygomatic osteotomy, and with sufficient care, they can all produce satisfactory results. The most crucial steps, however, are preoperative planning and intraoperative repositioning. Different incisions to access soft tissue enable osteotomy-related osseous anatomy visualization. Some surgeons refract the zygomaticomaxillary complex using lateral upper blepharoplasty, lateral eyebrow incision, and direct percutaneous approach, as well as intraoral, subtarsal, and transconjunctival approaches [7]. The coronal method, when combined with subciliary and intraoral approaches, considerably enhances zygomatic osteotomy. This technique enables the complete detachment of the zygoma from any external connections to soft tissues, which facilitates osteotomy and relocation. The zygomatic bone may be assimilated or infected following major soft tissue removal. However, this event is seldom observed in craniofacial surgery, where substantial amounts of bony structures are removed from their soft tissue connections and reattached. The line of osteotomy might be drawn along the original fracture site if it is apparent. This method may be employed only if the extent of the fracture is not too far posterior toward the orbital apex; nonetheless, it is applicable along the orbital floor.

The osteotomy can be extended along the inferolateral aspect of the anterior maxillary wall to the zygomatic buttress of the maxilla, where they intersect. Additionally, to complete the osteotomy, a few surgeons use an intraoral technique within the maxillary vestibule. This is a useful optional incision because, in some cases, bone grafts in the zygomatic buttress region may be required, particularly if nonrigid means of fixation are used along the orbital rims. Prior to any movement, a thorough examination of the zygomatic osteotomies is done to ensure that all osseous incisions have been made. The zygoma is relocated after it has been mobilized. If the malunion is severe, it is essential to remove some bone where the callus and new bone have grown in order to properly reduce the joint. The most effective locations for stabilizing the zygoma often involve the use of bone plates in the frontozygomatic and potentially infraorbital regions. Bony voids may need to have bone grafts placed into them. The defects in the lateral orbital wall are carefully fixed while the internal orbit is rebuilt. In instances where trauma has resulted in comminution of the zygoma, it may be necessary to apply an onlay graft or implant over the malar prominence to restore the natural contour [8].

The surgical approach to cicatricial ectropion is contingent on the conditions observed following the liberation of scar tissue pull in the lower lid region. Preoperative assessment of the cause and severity is crucial for successful treatment. Cicatricial ectropion can be treated in a variety of ways, including full-thickness skin grafts from preauricular or supraclavicular region, transposition flaps from the cheek, or flaps from upper eyelid. Z-plasty and V- to Y-plasty can also be performed [3]. The approach to surgically managing cicatricial ectropion is determined by the specific circumstances following the release of scar tissue traction in the lower lid area [9]. Correcting cicatricial ectropion can be accomplished in a number of ways, such as lengthening the anterior lamella with a transposition flap or full-thickness free skin grafts [10].

## Conclusions

In this case study, we have highlighted the significance of opting for an approach that guarantees optimal outcomes, prioritizing superior aesthetics while minimizing potential adverse effects. A specific surgical approach is needed for managing the issue of cicatricial ectropion. A thorough assessment of each case must be done for the desired management of post-traumatic facial anomalies. The surgeon determines the treatment approach, taking the patient's requirements into careful consideration.
